# Transactions of the Medical Association of Georgia, 34th Annual Session

**Published:** 1883-10-20

**Authors:** Eugene Foster

**Affiliations:** Chairman


### EDITORIALS AND MISCELLANEOUS.

### *THE TRANSACTIONS*

Of the Medical Association of Georgia, thirty fourth annual session, has been kindly handed us by Dr. J. A. Gray, Secretary.

At no period since the organization of the Society, has the volume of Transactions been so promptly issued as in this instance.

The work is very neatly gotten up, and contains 275 octavo pages. There are 254 names on the roll of membership. The names of deceased members recorded since the organization number 162, being a little more than four members per annum.

The next, or thirty-fifth annual meeting of the Association will take place at Macon the third Wednesday in April, 1884.

The papers published in the volume are as follows.

President's Address—By K. P. Moore, M. D.

Report on Diseases of Women for the First Congressional District—By K. J. Nunn, M. D.

Cerebro-Spinal Meningitis—Report on the Practice of Medicine for the Seventh Congressional District—By W. B. Wells, M. D.

Report of Section on Surgery for the Seventh Congressional District—By J. C. Bivings, M. D.

Report on Gynecology for the Seventh Congressional District—A Plea for the Education of Females—By Charles P. Gordon, M. D.

Climate—By A. Means, M. D., D.D., LL. D.

Hyosciamin in the Treatment of Violent Mania—By T. O. Powell, M.D.

Hotz's Operations for Entropion and Trichiasis, with fifteen cases—By A. G. Hobbs, M. D.

Report of Surgical Cases - By Thos. R. Wright, M. D.

Acute Inflammations of the Throat—By Charles W. Hickman, M. D.

The Dry Treatment of Suppurative Inflammation of the Middle Ear—By A. W. Calhoun, M. D.

Typhoid Fever—By L. G. Hardman, M. D.

Dysentery—By J. W. Duncan, M. D.

A Case of Circumcision—By B. R. Dostor, M. D.

Precautionary Measures and Contra-Indications to the use of Pressure by the Tampon in Diseases of the Pelvic Organs—By V. H. Taliaferro, M. D.

Taliaferro's Hard Rubber Intra-Uterine Steam Pessary, Retro-Displacement Pessary, Universal Pessary and Tracheloraphy—By G. H. Noble, M.D.

The Carbolic Acid and Iodine Treatment of Typhoid Fever—By Howard J. Williams, M. D.

Necrology—By Eugene Foster, M. D.

George Franklin Cooper, M. D.—By Eugene Foster, M. D.

William Morris Charters, M. D.—By Robert P. Myers, M. D.

John D. Fish, A. M., M. D.—By R J. Nunn, M. D.

Dr. Robert C. Carroll,—By A. Sibley Campbell, M. D.

The following are the officers elect for the present year :

President—A. W. Calhoun, Atlanta.

Vice-Presidents—R. J. Nunn, Savannah; M. P. Deadwyler, Elberton.

Secretary—James A. Gray, Atlanta.

Treasurer—E. C. Goodrich, Augusta

Orator—J. G. Hopkins, Thomasville.

BOARD OF CENSORS :

| Members. | Elected. |
|---|---|
| W. B. Wells, Red Clay | (1879.) |
| E. L. Connally, Atlanta | (1880.) |
| J. S. Todd, Atlanta | (1881.) |
| Eugene Foster, Augusta | (1882.) |
| A. W. Griggs, West Point | (1883.) |

The Special Committees are as follows :

*Committee on Inebriate Asylum.*—J. P. Logan, J. B. Baird, A. W. Calhoun, W. A. Love, W. O'Daniel

*Committee on Expert Testimony.*—H. V. M. Miller, J. T. Johnson, J. S. Todd, J. B. Baird.

*Committtee on Anatomical Material.*—H. V. M. Miller, T. S. Powell, Wm. Perrin Nicolson, W. S. Armstrong, DeSaussure Ford, J. B. Baird, G. G. Crawford, C. H. Hall, W. F. Westmoreland.

PRIZE ESSAYS FOR 1883–'84.

At the last meeting of the Medical Association of Georgia the Committee on Prize Essays was authorized to award a prize of fifty dollars to the best Essay on any subject connected with the practice of Medicine, Surgery, Obstetrics or Hygiene—provided one be found worthy of a prize.

Each Essay must be accompanied by a sealed packet, on which shall be written some motto or sentence, and within which shall be enclosed the author's name and residence. The same motto or sentence is to be written on the Essay to which the packet is attached.

Any clew by which the authorship of an Essay is made known to the Committee will debar such Essay from competition.

Competition for this prize is open to all regular physicians practicing in Georgia.

All Essays must be sent to Dr. Eugene Foster, Chairman of Committee on Prize Essays, 419 Broad street, Augusta, Georgia.

All Essays must be sent on or before March 10th, 1884.

In behalf of the Committee on Prize Essays,

EUGENE FOSTER, M. D., *Chairman.*

